# The Conditions Which Favor an Excessive Size of the Fœtus

**Published:** 1842-03

**Authors:** 


*The conditions which favour an excessive size of the Fœtus.*—Professor Osiander regards a too exclusive use for food, of articles composed of fecula, especially rye-bread, as contributing to produce a sort of fœtal hypertrophy; and he recommends to pregnant women who are obliged to live on such a diet, to abstain at regular times from food, and to take occasionally a saline purgative. The excessive developement of the fœtus is one of the causes of difficult labor, and the Professor relates five cases of this nature.—*American Journal of the Med. Sciences,* from *Zeitschrift fur gesammte Med.*

